# Full Embroidery Designed Electro-Textile Wearable Tag Antenna for WBAN Application

**DOI:** 10.3390/s19112470

**Published:** 2019-05-30

**Authors:** Bahaa Abbas, Salam K. Khamas, Alyani Ismail, Aduwati Sali

**Affiliations:** 1 Wireless and Photonics Research Centre of Excellence, Department of Computer and Communication Systems, Faculty of Engineering Universiti Putra Malaysia, Serdang 43400, Malaysia; alyani@upm.edu.my (A.I.); aduwati@upm.edu.my (A.S.); 2 The Electronic and Electrical Engineering Department, University of Sheffield, Sheffield S10 2TN, UK; s.khamas@sheffield.ac.uk

**Keywords:** WBAN, embroidery, electro-textile, ultra-high-frequency, RFID tags, antenna, body-centric, wearable

## Abstract

A flexible and totally wearable textile antenna is proposed by embroidering the conductive threads into garments. A purely polyester substrate has been utilized, which provides a tag that can be easily integrated with the clothes. The proposed tag antenna is small with dimensions of 72 × 20 × 2.75 mm^3^ and offers an enhanced performance in terms of gain and stability when worn on different body locations. Experimental results demonstrate an improved impedance matching owing to the elasticity of the E-shaped inductive feeder. Close agreement has been achieved between the simulated and measured results.

## 1. Introduction

Wireless body area networks (WBANs) have attracted much attention due to their wide range of applications in healthcare systems and rapid rescue services [1]. Wearable antennas represent essential elements for WBANs and, hence, there is a noticeable need for convenient and mechanically pliable wearable antennas that can be fabricated using textile materials [2]. This objective can be addressed by considering the significant advancements that have been made in electro-textiles fabrication over the last decade [3]. For instance, electro-textiles have been produced by embroidering conductive threads into a cloth when adopting conventional techniques that are used for general textiles [4,5]. Furthermore, electro-textiles have also been embroidered using computer-designed digital images [6]. However, in body-centric sensing systems, body-worn antennas are affected by a high dielectric constant and the resulting electrical conductivity and polarization properties, as well as by the absorptivity of human body tissues [1,5]. Such effects may deteriorate the radiative power and matching, as well as altering the antenna impedance with respect to that in free space [7]. Therefore, antenna-integrated clothing without a degraded performance represents a major issue facing the wide implementation of wearable antennas. Additionally, lightweight flexible textile materials that conform to the RF characteristics need to be employed in order to avoid the structural non-flexibility of traditional metals such as copper. Furthermore, durability represents another issue that needs careful consideration in the selection of wearable antenna materials due to environmental effects such as dirt, humidity, vulnerability to stretching, mechanical compression, and bending deformations. 

On the other hand, passive ultra-high-frequency (UHF) radio frequency identification (RFID)-based technology represents a promising choice as an energy-efficient wireless approach for future WBANs [1]. The major challenges of using E-textile RFID in body-centric areas are the flexibility of a full embroidery designed tag, system performance, as well as reliability in personal area networks (PANs) and WBANs. The open literature conclusively demonstrates a possibility for improving the RFID antenna design in order to achieve an enhanced performance when operating in a body-centric area and integrated with a garment. In addition, the presence of a human body has only been considered in a few published studies [1,4,5,8]. However, those RFID tag antennas are partially textile, since the non-textile EFDM or polytetrafluorethylene (PTFE) substrates that have been employed produced tags that were difficult to integrate with garments [9].

In this study, a novel and fully textile configuration is proposed, in which a purely polyester substrate is incorporated. Furthermore, a smaller-sized slotted inductively coupled feed with opposing E-shaped transmission lines has been proposed to improve the radiation characteristics when the tag is attached to the human body at different locations. The antenna performance has been evaluated experimentally and compared with those reported in earlier studies. Promising results in terms of gain and matching stability have been attained when the antenna is worn on the chest, arm, and head. This is in conjunction with a smaller size and easier garment integration. The tag antenna has been designed using the CST Microwave Studio full-wave electromagnetic simulator [10].

## 2. Antenna Design and Parametric Study 

**Figure 1:**
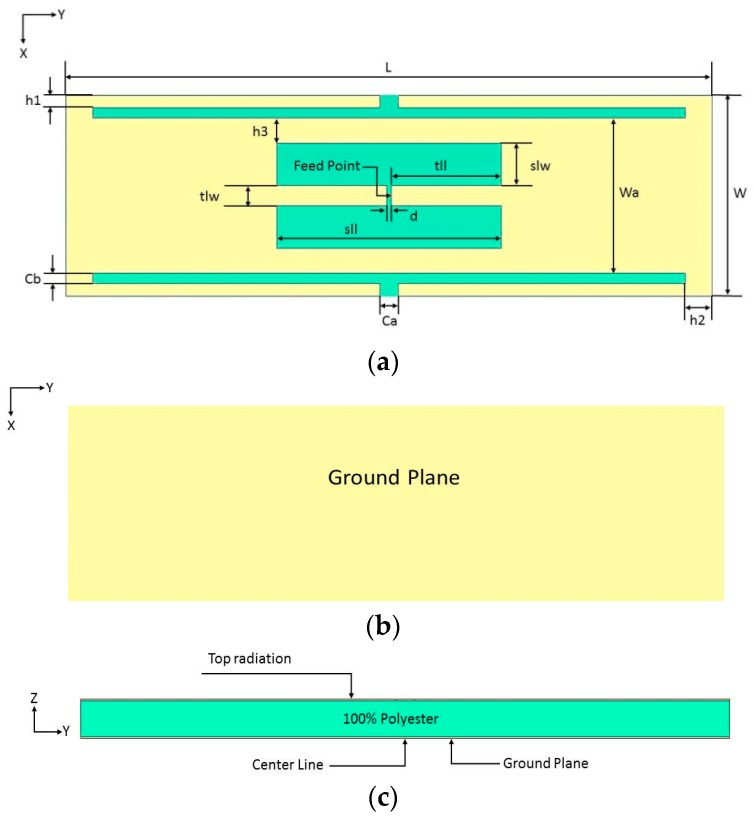
Proposed slotted tag antenna. (**a**) Top view, (**b**) ground plane, and (**c**) side view.

Figure 1 illustrates the slotted tag antenna configuration using the dimensions given in Table 1. The proposed antenna has been designed to operate at 915 MHz, and consists of two E-shaped feed transmission lines as well as a planar ground plane. A 100% polyester substrate with a thickness of 3 mm has been placed between, and directly attached to, the ground plane and the top-side metal surface. The substrate’s relative permittivity and loss tangent have been measured as 2.565 and 0.0025, respectively, by employing the free space method [11]. Additionally, an NXP UCODE G2iL SL3S1203 RFID tag chip with a size of 0.49 mm has been connected directly to both sides of the E-shaped center lines. The overall dimensions have been chosen as 74 × 20 × 3 mm^3^, and the operating frequency has been determined following an optimization process in order to achieve a conjugate impedance matching. 

The slot dimensions can be modified so as to achieve improved efficiency [12,13]. The antenna efficiency is affected by the patch transmission line. The fitting of the numerically computed input impedance to circuital expression was conducted as described in [14]. The degree of impedance matching between the chip and the antenna is given by the power transmission coefficient (PTC) τ as follows.

(1) $$\tau =\frac{4R_{c}R_{a}}{{\left|Z_{a}+Z_{c}\right|}^{2}},\;0\leq \tau \leq 1$$

τ is the power that can be delivered to the chip when $Z_{a}$ = $Z_{c}^{*}$, $R_{c}$ and $R_{a}$ denote the real impedance of the IC chip and proposed tag antenna, respectively, and $Z_{c}$ and $Z_{a}$ denote the complex impedance of the IC chip and antenna, respectively. Equation (1) indicates that appropriate conjugate impedance matching can provide the maximum transmission power for the antenna.

**Figure 2:**
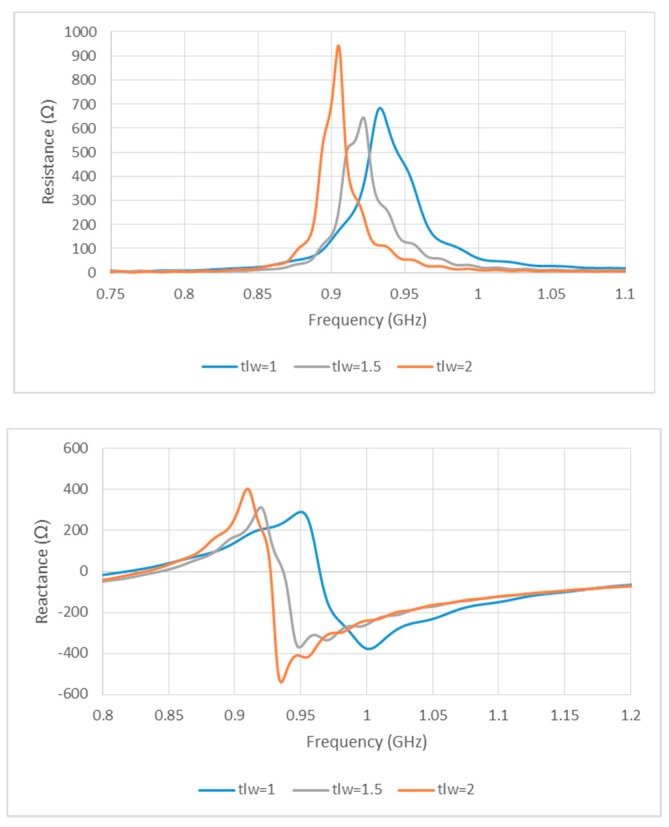
Simulated input impedance for various transmission line widths.

**Figure 3:**
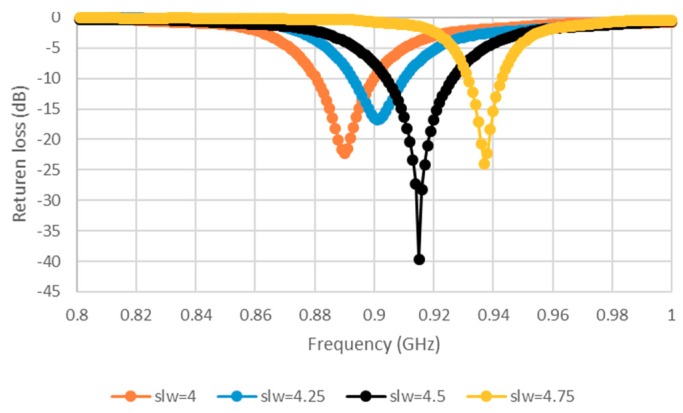
Simulated return losses (S11) for various slot widths (slw).

**Figure 4:**
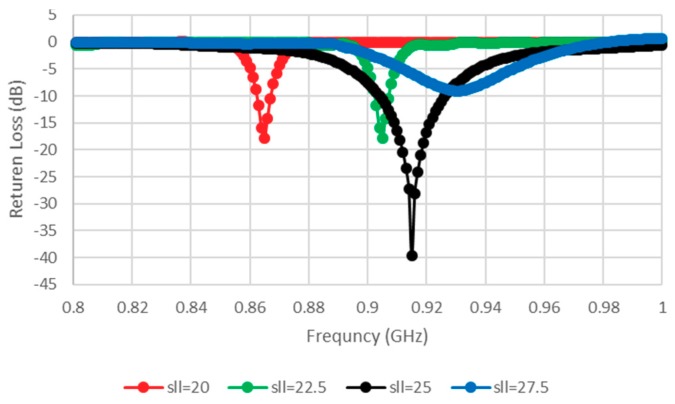
Simulated return loss (S11) for various slot lengths (sll).

**Figure 5:**
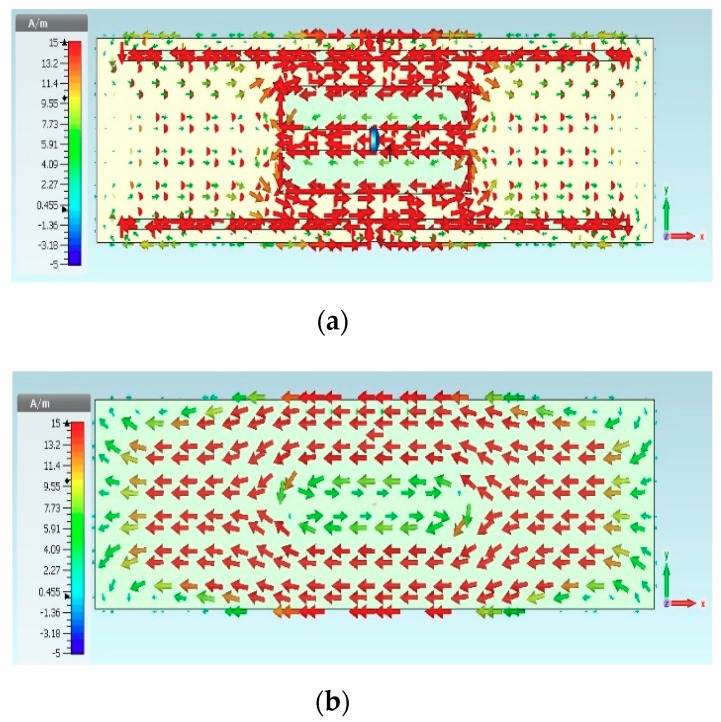
Current distributions of the proposed radio frequency identification (RFID) tag antenna. (**a**) Top surface and (**b**) ground plane.

The width of the slotted tag has been denoted as slw, while the distance between the opposite E-shaped feeds has been defined as sll. Conjugate matching can be achieved by varying the transmission line width, tlw, which affects the symmetrical E-shaped inductive feeders. The results are presented in Figure 2, where noticeable variations exist in the input impedance when the position and shape factor (which were chosen to synthesize the required complex input impedance for microchip matching here), and the cut and the slot parameters (like tlw and slw) of the matching slot are varied, which caused modifications by acting on the parameters. The antenna has an inductive resonance, which means the configuration provides conjugate matching to the capacitive impedance of the microchip. Therefore, tlw has been chosen as 2 mm. The impact of slot width (slw) variation has also been investigated, where it has been observed that the resistance and reactance are linearly and inversely proportional to tlw and slw, respectively. As a result, the antenna impedance increases, with a lower resonance frequency for a narrower slot, that is, when the slot moves closer to the folding. Therefore, the tag design may concentrate on optimizing the tlw (1, 1.5, and 2 mm) and slw (4, 4.25, 4.5, and 4.75 mm) parameters while the other dimensions are fixed, as shown in Figure 2 and Figure 3. Figure 3 portrays the minimization of return losses for an appropriate slw size and, similarly, when sll has been adjusted as illustrated in Figure 4. The parameters slw, sll, and tlw yield considerable effects on the current distribution since the magnitude of the top metal surface’s current has been maximized by employing the optimized parameters. As can be observed from Figure 5, the maximum current intensity occurs along the major edges. As a result, the system efficiency can be substantially improved due to the enhancement of radiation surrounding the edges of the antenna. In general, it is highly important to adjust the slotted dimensions for an optimum performance [15]. Similarly, the antenna performance and efficiency are affected by the E-shaped transmission lines and, hence, can be improved by modifying the slot dimensions [12,16]. As noted earlier, the high dielectric constant of the human body tissues reduces the radiated power and changes the impedance [5]. The tissue dielectric parameters have been calculated based on the 4-Cole–Cole model [17]. In the simulations, a statistical catalog of the human body has been used by considering three locations—head, chest, and arm—where the tag antenna has been placed [8]. In order to minimize losses, low electrical surface resistance needs to be ensured for fabrics, thus improving the antenna performance [18]. It is well known that silver-plated threads exhibit higher conductivity, which minimizes conductive losses in transmission lines, antennas, electro-textile resonators, and other microwave devices [19]. In this study, Syscom Advanced Materials with silver-plated nylon fibers (Agsis™, 1305 Kinnear Rd, Columbus, Oh, 43212, United State) have been employed, where the fiber material was polyamide 6 and the outer metallization layer was silver [18]. The used conductive thread has the following properties: conductivity of 29 × 10^6^ s/m, DC resistance of 26 ± 6 Ω/m, weight of 0.007 ± 0.00075 g/ft, maximum operating temperature of 95 °C, and a melting point of 215 °C. As mentioned previously, with recent advancements, electro-textiles have been embroidered using computer designated technical images [7]. The conductive tag parts have been embroidered on the nonconductive polyester substrate. The embroidery process involved 3419 and 4320 stitches for the ground plane and top metal surface, respectively. The aesthetic shapes and tag design of the back-side antennas are digitized and programmed for the Tajima Embroidery machine [20,21]. The antenna substrate has been attached to the electro-textile using a vacuum pack that has compressed the substrate height to 2.75 mm. The compression process minimizes the air gaps between layers, which improves the characterization accuracy of the electro-textile. In addition, a flexible glue has been employed, and the plastic packaging can be removed when the tag is integrated to the garment. It should be noted that the variation in the substrate thickness shifts the resonance frequency to 970 MHz. However, since the RFID operates closer to the human body, then the high-permittivity of human tissues can shift the resonance back to the desired range. The RFID microchip, with a Pi ”Power Input” (min) of −18 dBm, has been attached to the antenna using a silver-based conductive adhesive with an electrical resistivity of less than 1 × 10^−4^ Ω. The embroidered textile permanently results in an anisotropic pattern in which the conductivity is strongly dependent on the direction of the current flow, geometry, and stitching density of the pattern, which gives high performance [4,22].

## 3. Measurements and Results

**Figure 6:**
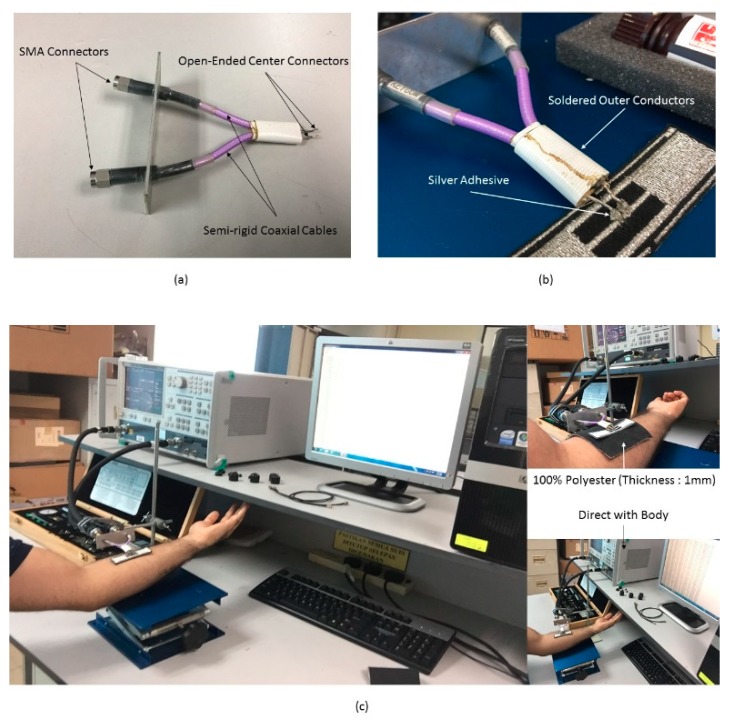
(**a**) Differential probe. (**b**) Open-ended side of semi-rigid cables. (**c**) Measurement setup for tag antenna.

**Figure 7:**
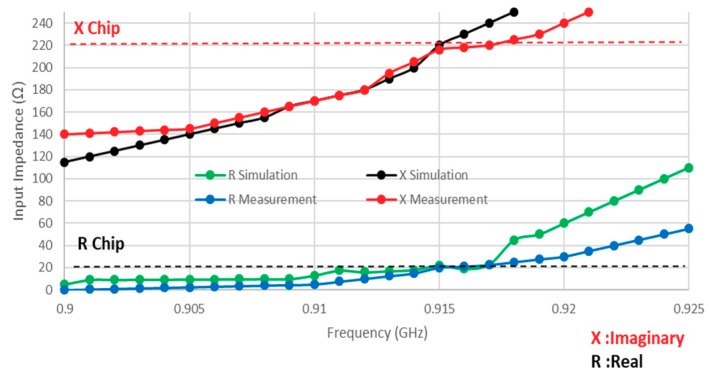
Input impedance of an antenna on a human body.

As mentioned earlier, a purely polyester substrate has been employed. A differential probe has been used to measure the input impedance of the balanced antenna as well as the return losses [19,23]. Measurements have been conducted in a normal environment and differential probes have been connected to the vector network analyzer and RFID tag antenna from each end and attached to a human arm. The measured results indicate that the chosen positions in Figure 6, direct attachment, and garment thickness of 1 mm, have no impact on the tag antenna performance. The input impedance of a tag antenna located around a human body is illustrated in Figure 7, with reasonable agreement between measured and simulated impedances that are close to the designed chip impedance of 23−j224 Ω at 915 MHz. However, some discrepancies can be observed between the two datasets that may be attributed to the soldering on the measuring probe, mismatch between the feeding lines and the SMA “SubMiniature version A” connectors, defects during the fabrication process of the embroidery machine, as well as the presence of uneliminated air gaps between the antenna layers. The main performance enhancements depend on the small slot cuts in the top radiation part that add inductance to the patch elements. The chip impedance lumped-element values were viewed with 23−j224 Ω CST Microwave Studio software. 

**Figure 8:**
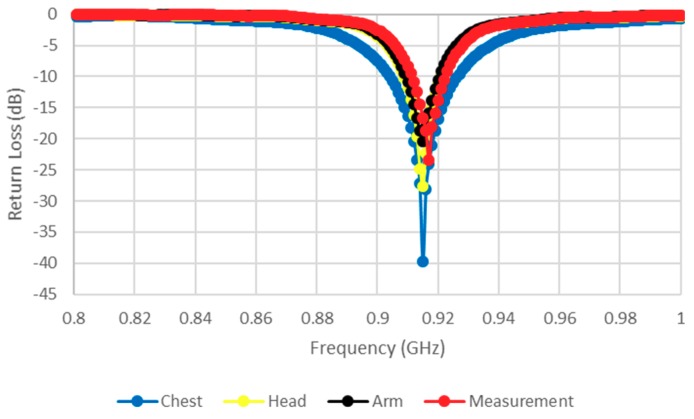
Return losses at three different locations on human body.

**Figure 9:**
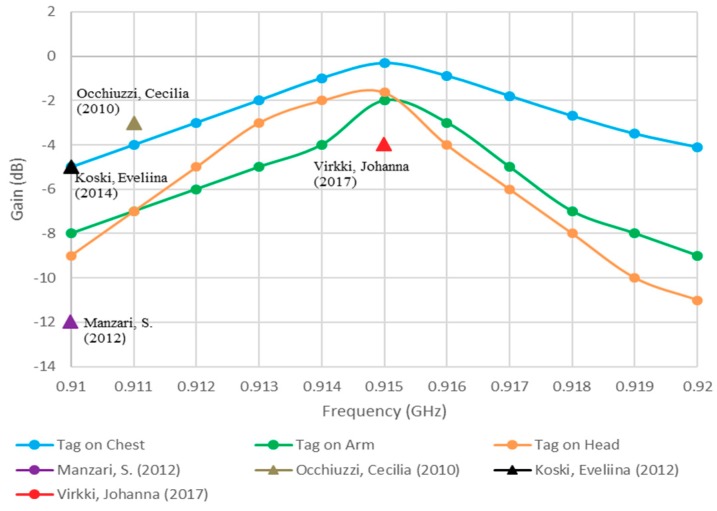
Simulated gain when the proposed antenna is fixed at various body locations compared to earlier studies.

The return losses have been monitored at three locations on the human body—chest, arm, and head—as illustrated in Figure 8, where it can be observed that a stable and excellent performance has been accomplished with good agreement between experimental and simulated results. It has been assumed that there is no variation in the real part of the chip impedance over the considered frequency [24]. Figure 9 illustrates the simulated gain when the antenna is attached at various body locations, where it can be noted that the gain increases notably when the antenna is attached to the chest. This can be understood by noting that the human body acts as a reflector that blocks backward radiation while increases the forward gain and directivity. Therefore, an improved antenna performance can be achieved if the antenna is attached to a larger body area, such as the chest. The simulated gain ranges from −0.325 dBi on the chest to −2 dBi on the head and arm. The maximum gain and impedance matching show a minute responsiveness to the location on the body because of the availability of the ground plane.

**Figure 10:**
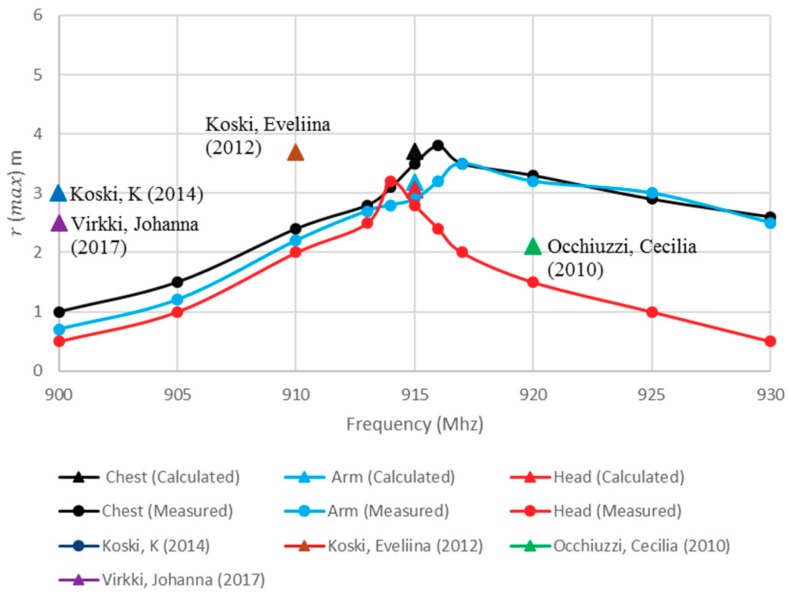
Measured and calculated read range when the tag antenna is fixed at various body locations compared to earlier studies.

**Figure 11:**
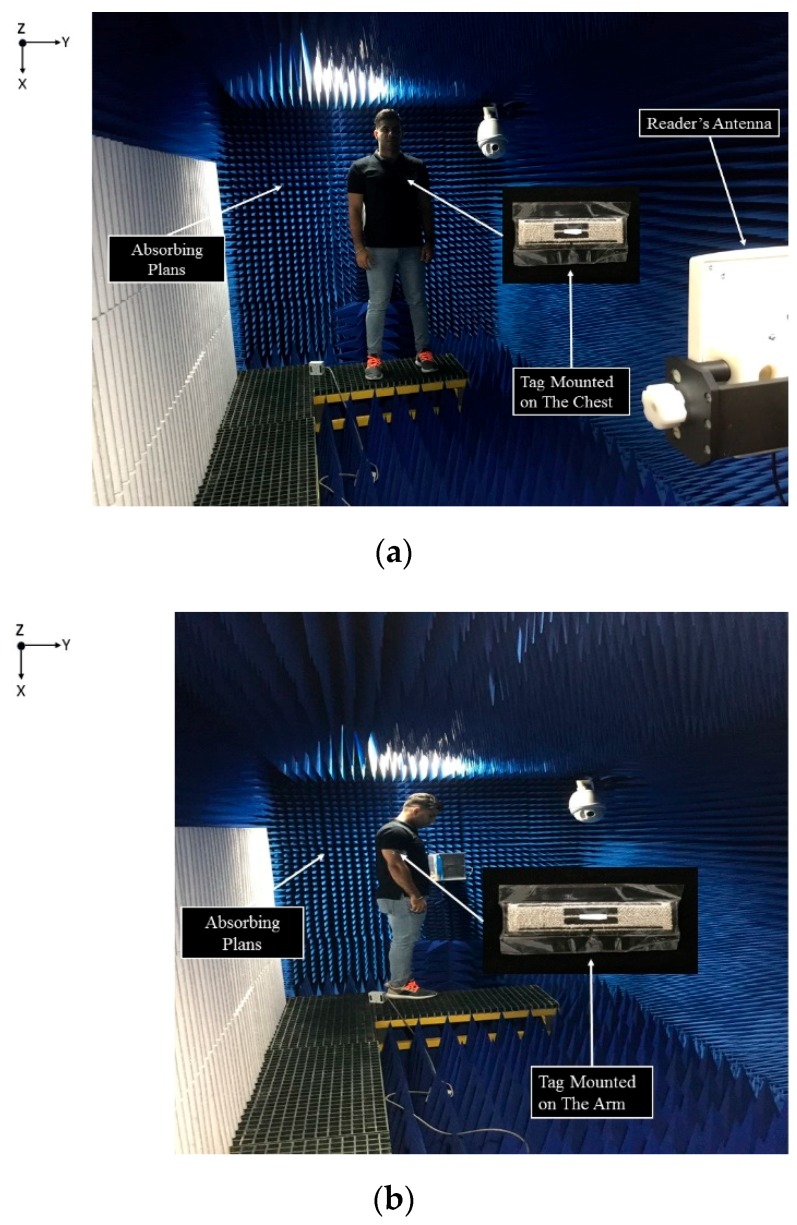

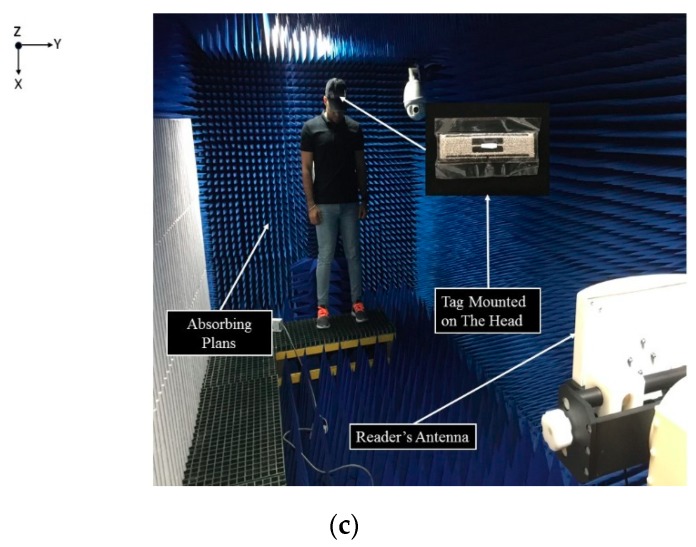
Reading distance of antenna when located on (**a**) chest, (**b**) arm, and (**c**) head as measured in the +z direction.

The maximum reading distance, r_max_, of the proposed antenna can be calculated as Equation (2) [25].

(2) $$r_{max}=\frac{\lambda }{4\pi }\sqrt{\frac{EIRPG_{r}}{P_{th}}},$$

where λ/4π = 0.26 m, EIRP denotes the effective isotropically radiated power of 3.45 W, $P_{th}$ is the threshold power of −17.6 dBm, and $G_{r}$ is the tag antenna gain. The ability of the tag to communicate with the reader determines the actual reader-body distance which, in turn, defines the tag’s reading region. The reading range has been measured using GAO UHF Gen2 RFID [26] reader at eleven frequency points over the UHF band. Figure 10 shows good agreement of the measured and calculated reading distances of the tag antenna when compared to published data, thus validating the presented tag antenna design. In addition, it can be observed that the calculated and measured read ranges are in close agreement, which further validates the proposed configuration. The reading distance also depends on the location, since shadows may arise from the shape of each body segment and the process of their absorption. In order to further investigate this issue, tags have been attached at two locations on a T-shirt at the arm and chest, and on a cap for the head in Figure 11. Basically, it is difficult to attach an electro-textile tag accurately at identical locations, especially if the tags are bent on the body surface, such as on the chest, where the uncertainty of the model would be more significant. However, the simulation and measurement confirmed the reliability of the proposed design with respect to the reading performance. Additionally, the used antenna components are unique, since they are totally flexible and fully textile.

## 4. Conclusions 

A novel fully textile UHF RFID tag antenna has been developed that can be easily integrated with clothes. This is an electrically small antenna with the dimensions of 72 × 20 × 2.75 mm^3^. Experimental work demonstrated an excellent performance, with a higher gain of −0.325 dB and a reading distance of 3.8 m when the antenna is worn. The designed antenna has a low fabrication cost using a purely polyester substrate and embroidery fabrication process. In addition, commercial materials that are available in the market have been employed. The conductive and dielectric materials have been studied and tested in order to demonstrate the improved performance compared to published configurations as demonstrated in Table 2 and Figure 9. The presented design is suitable for body-centric systems, biomedical as well as other wearable antenna applications.

On the other hand, reductions in gain and reading distance have been observed when the antenna is displaced from the chest, which could be due to the difference in biological tissues that dissipate energy and exhibit high dielectric constants, as well as the larger reflecting chest size. This restricts the radiation efficiency and changes the antenna impedance compared to the free-space counterpart. The presented work can be extended to consider the impact of human body curvature on the antenna performance. Another improvement aspect is to study the effects of human body movement dynamics on the antenna radiation characteristics.

## Figures and Tables

**Table 1:** Antenna dimensions.

Parameters	Dimensions (mm)
Antenna side length (L)	74
Antenna side width (W)	20
Internal side width (Wa)	15.5
Slotted width (slw)	4.25
Slotted length (sll)	25
Transmission line length (tll)	12.25
Transmission line width (tlw)	2
Feed point (d)	0.49
The cut between outer line (Ca)	2
The cut line (Cb)	1
The outer line width (h1)	1
The distance between the cut and side edge (h2)	4
The distance between the cut (h3)	2.5

**Table 2:** Comparison of various body centric antenna properties.

Reference	Dimensions (λeff3)	Substrate	Patch & GP	Reading Distance (m)
[1]	0.22 × 0.12 × 0.015	Flexible	Textile	3–4
[4]	0.5 × 0.3 × 0.01	Flexible	Textile	3.9
[8]	0.37 × 0.25 × 0.016	Flexible	Copper	2.1
[27]	0.51 × 0.07 × 0.0005	Flexible	Copper	2.5
Proposed Tag	0.28 × 0.08 × 0.011	Textile	Textile	3.8
